# Identification and Full Characterisation of Two Novel Crustacean Infecting Members of the Family *Nudiviridae* Provides Support for Two Subfamilies

**DOI:** 10.3390/v13091694

**Published:** 2021-08-26

**Authors:** Kelly S. Bateman, Rose Kerr, Grant D. Stentiford, Tim P. Bean, Chantelle Hooper, Benigna Van Eynde, Daan Delbare, Jamie Bojko, Olivier Christiaens, Clauvis N. T. Taning, Guy Smagghe, Monique M. van Oers, Ronny van Aerle

**Affiliations:** 1 International Centre of Excellence for Aquatic Animal Health (ICOE AAH), Centre for Environment, Fisheries and Aquaculture Science (Cefas), Barrack Road, The Nothe, Weymouth, Dorset DT4 8UB, UK; rose.kerr@cefas.co.uk (R.K.); grant.stentiford@cefas.co.uk (G.D.S.); chantelle.hooper@cefas.co.uk (C.H.); ronny.vanaerle@cefas.co.uk (R.v.A.); 2 The Roslin Institute and Royal (Dick), School of Veterinary Studies, Easter Bush Campus, The University of Edinburgh, Midlothian EH25 9RG, UK; tim.bean@roslin.ed.ac.uk; 3 Department of Plants and Crops, Faculty of Bioscience Engineering, Ghent University, 9000 Ghent, Belgium; benignavaneynde@gmail.com (B.V.E.); olchrist.Christiaens@UGent.be (O.C.); TiziClauvis.TaningNji@UGent.be (C.N.T.T.); guy.smagghe@ugent.be (G.S.); 4 Animal Sciences Unit, Fisheries, Flanders Research Institute for Agriculture, Fisheries and Food (ILVO), 8400 Ostend, Belgium; daan.delbare@ilvo.vlaanderen.be; 5 School of Health and Life Science, Teesside University, Middlesbrough TS1 3BA, UK; J.Bojko@tees.ac.uk; 6 Laboratory of Virology, Plant Sciences Group, Wageningen University and Research, P.O. Box 16, 6700 AA Wageningen, The Netherlands; monique.vanoers@wur.nl

**Keywords:** nudivirus, *Crangon crangon*, *Carcinus maenas*, brown shrimp, shore crab, virus classification, core genes, Crangon crangon nudivirus (CcNV), Carcinus maenas nudivirus (CmNV), bacilliform virus, taxonomy

## Abstract

Multiple enveloped viruses with rod-shaped nucleocapsids have been described, infecting the epithelial cell nuclei within the hepatopancreas tubules of crustaceans. These bacilliform viruses share the ultrastructural characteristics of nudiviruses, a specific clade of viruses infecting arthropods. Using histology, electron microscopy and high throughput sequencing, we characterise two further bacilliform viruses from aquatic hosts, the brown shrimp (*Crangon crangon*) and the European shore crab (*Carcinus maenas*). We assembled the full double stranded, circular DNA genome sequences of these viruses (~113 and 132 kbp, respectively). Comparative genomics and phylogenetic analyses confirm that both belong within the family *Nudiviridae* but in separate clades representing nudiviruses found in freshwater and marine environments. We show that the three thymidine kinase (*tk*) genes present in all sequenced nudivirus genomes, thus far, were absent in the Crangon crangon nudivirus, suggesting there are twenty-eight core genes shared by all nudiviruses. Furthermore, the phylogenetic data no longer support the subdivision of the family *Nudiviridae* into four genera (*Alphanudivirus* to *Deltanudivirus),* as recently adopted by the International Committee on Taxonomy of Viruses (ICTV), but rather shows two main branches of the family that are further subdivided. Our data support a recent proposal to create two subfamilies within the family *Nudiviridae*, each subdivided into several genera.

## 1. Introduction

Few viruses infecting marine invertebrates have been formally characterised with most tentatively assigned to families based upon morphological, developmental and replicative characteristics within the host cell [1]. This was largely due to the lack of crustacean cell lines for culturing viral infections, but with the recent development and increasing availability of high-throughput sequencing technologies comprehensive descriptions now facilitate classifications and taxonomic placement of novel viruses, at least to family level [2,3]. Using these approaches, several previously unclassified crustacean viruses have been assigned to the family *Nudiviridae* [4].

Nudiviruses infect a wide array of arthropods and exhibit nuclear replication. They have double-stranded, circular DNA genomes ranging in size between 96 and 232 kbp. Their virions are enveloped and contain rod-shaped nucleocapsids [4,5,6,7]. Although gene order is poorly conserved among nudivirus genomes [4], to date 31 core genes have been identified as being shared amongst all members of the *Nudiviridae*, including homologs of several baculovirus core genes [8,9]. Viruses now classified in the family *Nudiviridae* have previously been named ‘non-occluded baculoviruses’ [10] and ‘intranuclear bacilliform viruses’ [11]. However, nudiviruses have been shown to form a distinct lineage separate from the baculoviruses [4], despite sharing a set of core genes and several ultrastructural features. The family *Nudiviridae* was initially named to reflect the lack of viral occlusion bodies (‘nudi-’meaning bare), differentiating these non-occluded viruses from the occluded baculoviruses. However, there are now several examples of viruses classified in the family *Nudiviridae*, based on gene content and phylogeny, for which occlusion bodies have been observed and where genes encoding (structural) homologs of the baculovirus polyhedrin protein have been identified [8,12,13]. In addition, endogenous viral elements (EVEs) derived from nudiviruses have been reported to be incorporated into the genomes of multiple arthropod species [14,15,16,17].

The subdivision of the family *Nudiviridae* into four genera has recently been approved by the International Committee on Taxonomy of Viruses (ICTV) and will be published later this year (ICTV release 2021). The genera *Alphanudivirus* and *Betanudivirus* contain species affecting insect hosts. *Penaeus monodon nudivirus* (PmNV) was the first aquatic species to be placed within the *Nudiviridae* [12] and is now grouped with *Homarus gammarus nudivirus* (HgNV) [18] within the genus *Gammanudivirus*. Isolates of these two viruses were found in aquatic hosts from marine environments. A virus infecting cranefly larvae [8] belongs to the species *Tipula oleracea nudivirus* (ToNV) and was assigned to the genus *Deltanudivirus*. A third aquatic virus, Dikerogammarus haemobaphes nudivirus (DhNV) infecting a peracarid host from a freshwater environment was also tentatively placed within the family *Nudiviridae,* but has not yet been formally assigned to a genus. Due to the low level of similarity between the encoded proteins predicted for DhNV, when compared to members of the *Gammanudivirus* and *Deltanudivirus*, Allain et al. [19] proposed the erection of the additional genus *Epsilonnudivirus* to contain peracarid-infecting nudiviruses.

As highlighted previously [1,19], it is very likely there are additional nudiviruses infecting aquatic crustacean hosts from both marine and freshwater systems. Virome analysis of the European brown shrimp *Crangon crangon* identified sequences homologous to nudiviruses that formed three large contigs, but which could however not be assembled into a full genome sequence [20]. Crangon crangon bacilliform virus (CcBV) has been described in the brown shrimp, caught in the Clyde Estuary, UK [21]. The infection was initially described as an intranuclear bacilliform virus, owing to the ultrastructure, morphology and size of the virions [21]. This virus targets the hepatopancreatic epithelial cells, and infected cells display hypertrophied nuclei with marginalised chromatin and an eosinophilic inclusion body. The rod-shaped nucleocapsids are enveloped with a characteristic bulb shaped protuberance of the envelope at one end and measure 280 nm × 72 nm [21]. The virus has since been found to be ubiquitous within this species in European waters [22,23]. Similarly, Carcinus maenas bacilliform virus (CmBV) was described infecting the European shore crab from the Clyde and Tyne estuaries in the UK [23]. The virus also targets the hepatopancreatic epithelial cells and displays pathology comparable to that described for CcBV. CmBV has since been identified affecting crabs in both their native ranges in Northern Europe and invasive ranges in Atlantic Canada [24]. We re-isolated CcBV and CmBV from shrimp and crab tissues sampled in UK and Canadian waters, respectively, to enable in depth characterisation of these viruses.

Here, our aim was to collect histological, ultrastructural and genomic data from bacilliform viruses infecting the two aquatic crustacean species, *C. crangon* and *C. maenas*, and compare these characteristics to what is known from nudivirus infections from terrestrial and aquatic environments. First, collected samples of both crustacean species were analysed using histology and electron microscopy to identify the infection and analyse the virions morphologically. Next, genome sequencing and de novo assembly was carried out to determine the genome structure, the presence/absence of the nudivirus core genes and to compare related viruses using phylogenetics. Utilizing the new latinized binominal method for the naming of virus species, we propose the virus species *Gammanudivirus* c*racrangoni* and *Gammanudivirus camaenasi*, with the common names Crangon crangon nudivirus (CcNV) and Carcinus maenas nudivirus (CmNV), respectively, to be used in the rest of this manuscript.

## 2. Materials and Methods

### 2.1. Sample Collection

*Crangon crangon* specimens were caught off the coast of Belgium as described by Van Eynde et al. [22]. *Carcinus maenas* were collected from the shoreline in Canada as described by Bojko et al. [24]. Hepatopancreas samples were dissected from *C. crangon* and *C. maenas* samples and fixed in Davidson’s sea water fixative for histology and 2.5% glutaraldehyde in 0.1 M sodium cacodylate buffer for electron microscopy. Hepatopancreas from each animal was also dissected for molecular analysis, *C. crangon* were snap frozen in liquid nitrogen and stored at −80 °C and samples from *C. maenas* were fixed in 100% ethanol.

### 2.2. Histology

Samples were fixed in Davidson’s sea water fixative for a minimum of 24 h before samples were transferred to 70% industrial methylated spirit (Ethanol and Methanol mixture, Pioneer Research Chemicals Ltd., Colchester, UK). Fixed samples were processed to wax in a vacuum infiltration processor (Leica Peloris) using standard protocols. Sections were cut at a thickness of 3–5 µm on a rotary microtome and were mounted onto glass slides before staining with haematoxylin and eosin (H&E). Stained sections were analysed by light microscopy (Nikon Eclipse E800) and digital images were taken using the Lucia™ Screen Measurement System (Nikon, Surbiton, UK).

Samples were analysed for presence of viral inclusions within hypertrophied nuclei of the epithelial cells within the hepatopancreas tubules. A grading scheme (Grade 0 to Grade 4) as described by Stentiford and Feist [23] was used to determine high and low prevalence of the viral infections, for Grade 0 the viral infection appeared to be absent from the histology section whereas Grade 4 described that most cells within the hepatopancreatic tubules showed infection.

### 2.3. Transmission Electron Microscopy (TEM)

Hepatopancreas tissue was fixed in 2.5% glutaraldehyde (Agar Scientific, Stansted, UK) in 0.1 M sodium cacodylate buffer (pH 7.4) (Agar Scientific, Stansted, UK) for a minimum of 2 h at room temperature and rinsed in 0.1 M sodium cacodylate buffer (pH 7.4). Tissues were post-fixed for 1 h in 1% osmium tetroxide (Agar Scientific, Stansted, UK) in 0.1 M sodium cacodylate buffer. Samples were washed in three changes of 0.1 M sodium cacodylate buffer before dehydration through a graded acetone series. Samples were embedded in Agar 100 epoxy (Agar Scientific, Agar 100 premix kit medium) and polymerised overnight at 60 °C in an oven. Semi-thin (1–2 µm) sections were stained with Toluidine Blue for viewing with a light microscope to identify suitable target areas. Ultrathin sections (70–90 nm) of these areas were mounted on uncoated copper grids (Agar Scientific, Stansted, UK) and stained with 2% aqueous uranyl acetate (Agar Scientific, Stansted, UK) and Reynolds’ lead citrate [25]. Grids were examined using a JEOL JEM 1400 transmission electron microscope and digital images captured using an Advanced Microscopy Techniques (AMT) XR80 camera and AMT V602 software.

### 2.4. DNA Extraction

Total DNA was extracted from hepatopancreas tissue samples of *C. crangon* using the DNeasy Blood and Tissue kit (Qiagen) DNA extraction kit (using manufacturer’s instructions) in preparation for Illumina sequencing. Additionally, DNA was also extracted from these tissue samples for Nanopore sequencing using an adapted phenol:chloroform:isoamyl alcohol (PCI) method (25:24:1). Roughly 10 mg of tissue was added to 900 µL of Lifton’s buffer (100 mM EDTA, 25 mM Tris-HCl pH 7.5, 1% SDS) with Proteinase K (0.2 mg/mL end concentration). Samples were homogenised very gently with a pellet pestle for 30 s and incubated at 56 °C overnight. One hundred microlitres of 5 M potassium acetate were added and mixed by gentle inversion prior to a 30 min incubation on ice and a 10 min centrifugation at 10,000 rpm. DNA was extracted from this supernatant with an equal volume of PCI and cleaned with a two-step ethanol precipitation. All mixing was done by gentle inversion and pellet resuspension was completed overnight in TE buffer without agitation [26]. DNA was quantified by Quantus fluorometer (Promega, UK) and checked by gel electrophoresis. Unless otherwise stated all chemicals were provided by Sigma-Aldrich (Gillingham, UK). The latter extraction method was also used to extract DNA from hepatopancreas tissue samples of *C. maenas*.

### 2.5. DNA Library Construction and Sequencing

DNA sequencing libraries were prepared for Illumina sequencing using the Nextera XT library preparation kit (Illumina, San Diego, CA, USA) and sequenced on an Illumina MiSeq using V3 chemistry (Illumina; 2 × 150 bp for *C. crangon* (5 individual samples pooled) and 2 × 300 bp for *C. maenas* (single sample)). A Nanopore sequencing library was constructed using the ligation sequencing kit SQK-LSK109 and the native barcoding kit (EXP-NBD103) for 5 *C. Crangon* samples (Oxford Nanopore Technologies Ltd., Oxford, UK). The barcoded DNA samples were pooled in equimolar concentrations and the prepared library loaded onto a SpotON flowcell R9.4.1 (FLO-MIN106) and sequenced for 11 h on a MinION device (Oxford Nanopore Technologies). Data were base-called and demultiplexed locally on a laptop using Albacore 2.3.1 (base-calling software released by Oxford Nanopore Technologies).

### 2.6. Sequence Assembly

Illumina reads obtained for the *C. maenas* and *C. crangon* samples were quality-trimmed using Fastp v0.20.0 ([27]; default parameters) and normalised using BBnorm which is part of the BBMap suite ([28]; default parameters). The quality-trimmed normalised Illumina reads of the *C. maenas* sample were assembled de novo using Unicycler v0.4.8 ([29]; using the --no_correct parameter). For the *C. crangon* sample, all Nanopore reads were combined into a single fastq file, followed by demultiplexing and adapter and barcode sequence removal using Porechop v0.2.3 (default parameters) [30]. A hybrid de novo genome sequence assembly was performed using a combination of the quality-trimmed normalised Illumina and Nanopore reads with Unicycler v0.4.8 (using the --no_correct parameter). Resulting assembled contig sequences were submitted to similarity searches using blastn v2.9.0+ [31] and the NCBI nucleotide database (accessed on 5 July 2020) to identify potential viral sequences.

Sequence statistics and general manipulation of fasta/fastq files were performed using SeqKit v0.11.0 [32]. All reads for each sample were mapped to the corresponding assembled CmNV or CcNV genome sequences using Minimap2 2.17-r941 [33]. SAMtools v1.9 [34] was used to convert the resulting SAM files into BAM format and to sort and index these prior to statistical analysis and visualisation of the mapping results using QualiMap v2.2.2 [35]. The genome sequences were screened for tandem repeats using the Tandem Repeats Finder tool v4.09 ([36]; default settings and alignment score > 100).

### 2.7. Gene Prediction and Annotation

Open reading frames were predicted using a selection of gene prediction tools, including Prokka v1.14.0 ([37]; default settings and --kingdom Viruses), fgenesv0 (http://www.softberry.com, accessed on 3 September 2020; standard code and circular sequence), GenemarkS [38]; using both the Intronless eukaryotic and Virus sequence types and genetic code 11, and Vgas [39]; using ATG as start codon type). Putative protein sequences were further analysed when they were predicted by two or more tools. The protein sequences were annotated using blastp v2.9.0+ sequence similarity searches ([40]; E-value cut off 0.001) against a reference database of nudivirus protein sequences including PmNV (KJ184318.1), Homarus gammarus nudivirus (HgNV; MK439999.1), Dikerogammarus haemobaphes nudivirus (DhNV; MT488302.1), Gryllus bimaculatus nudivirus (GbNV; NC_009240.1), Heliothis nudivirus 1 (HzNV-1; AF451898.1), HzNV-2 (NC_004156.2), Oryctes rhinoceros nudivirus (OrNV; NC_011588.1), ToNV (NC_026242.1), Drosophila innubila nudivirus (DiNV; NC_040699.1) and a further 4 Drosophila melanogaster nudiviruses [Mauternbach virus (MNV; MG969167), Kallithea virus (KNV; NC_033829.1), Tomelloso virus (TNV; NC_040789.1) and Esparto virus (ENV; NC_040536.1)] with the addition of shared Autographa californica multiple nucleopolyhedrovirus (AcMNPV; NC_001623.1) proteins as outgroup. InterProScan v5.31-70.0 (using the following databases: CDD-3.16, Coils-2.2.1, Gene3D-4.2.0, Hamap-2018_03, MobiDBLite-2.0, PANTHER-12.0, Pfam-31.0, PIRSF-3.02, PRINTS-42.0, ProDom-2006.1, ProSitePatterns-2018_02, ProSiteProfiles-2018_02, SFLD-4, SMART-7.1, SUPERFAMILY-1.75, TIGRFAM-15.0) was used to search for conserved protein motifs/domains.

### 2.8. Gene Orthology and Nudivirus Core Gene Analysis

Comparative proteome analyses across all nudiviruses, including the two newly sequenced viruses, were performed by blastp v2.9.0+ sequence similarity searches (E-value < 0.001) using the protein sequences of each virus and the nudivirus protein reference database described above. OrthoFinder v2.3.11 ([41]; parameters: -A muscle -M msa -T raxml) was used to identify orthologous groups of proteins (orthogroups) in the proteomes of all of the nudiviruses and AcMNPV (NC_001623.1) as outgroup. Reciprocal BLAST searches were conducted using the protein sequences of all nudiviruses (blastp v2.9.0+; evalue cut-off 0.001; [40]) and the results were used together with the orthologous genes identified by OrthoFinder to identify core nudivirus genes. Genome maps showing the position and orientation of nudivirus core genes were created using the R packages gggenes (available at https://github.com/wilkox/gggenes; accessed on 13 February 2021) and ggplot2 [42] in RStudio v1.2.1335 [43]. To allow for better comparisons, the genome sequences were rearranged, such that all linear representations of the viral genomes started with the DNA polymerase gene.

### 2.9. Phylogenetic Analysis

Proteins within each orthogroup were aligned using MAFFT v7.455 ([44]; parameters used: --localpair --maxiterate 1000) and poorly aligned positions and divergent regions were removed using Gblocks v0.91b [45]. Sequences were converted to PHYLIP format (http://sco.h-its.org/exelixis/resource/download/software/fasta2relaxedPhylip.pl; accessed on 15 April 2021) followed by best model selection (https://cme.h-its.org/exelixis/resource/download/software/ProteinModelSelection.pl; accessed on 15 April 2021), prior to rapid Bootstrap analysis (1000 bootstraps) and search for best-scoring ML tree using RaxML v8.2.12 [46]. The gene trees were pruned using PhyloTreePruner v1.0 [47] to remove paralogues and trees containing less than 12 species. Pruned orthogroups were aligned using MAFFT v7.455 (--auto –reorder), trimmed using Trimal v1.2 ([48]; parameter used: -automated1) and concatenated using a perl script (https://github.com/nylander/catfasta2phyml; accessed on 1 December 2019). The resulting supermatrix of concatenated orthologous protein sequences was analysed with RaxML v8.2.12 [46] using a PROTGAMMAAUTO amino acid substitution model and 1000 bootstraps to generate a final phylogenetic tree. A separate phylogenetic analysis was conducted (as described above), but using nudivirus protein sequences only (i.e., without AcMNPV) and concatenated orthologous protein sequences. Phylogenetic trees were drawn using Figtree v1.4.4 [49] and images representing the various species and duplication events identified by OrthoFinder were added in Inkscape v0.91 (https://inkscape.org; accessed on 1 May 2018).

Figures representing the fully identified viral genome sequences were generated by combining gene orientation and annotation layers generated by ApE v2.0.55 (https://jorgensen.biology.utah.edu/wayned/ape/; accessed on 7 July 2019) with various layers showing GC content, GC skew, position of tandem repeats and sense and antisense genes produced by Circa (http://omgenomics.com/circa; accessed on 7 July 2019). Inkscape v0.91 (https://inkscape.org; accessed on 1 May 2018) was used to combine these layers into single genome figures. GC content and GC skew were calculated over 100 bp windows using a perl script (https://github.com/DamienFr/GC_content_in_sliding_window; accessed on 6 July 2019).

## 3. Results

### 3.1. Histological and Ultrastructural Observations

**Figure 1:**
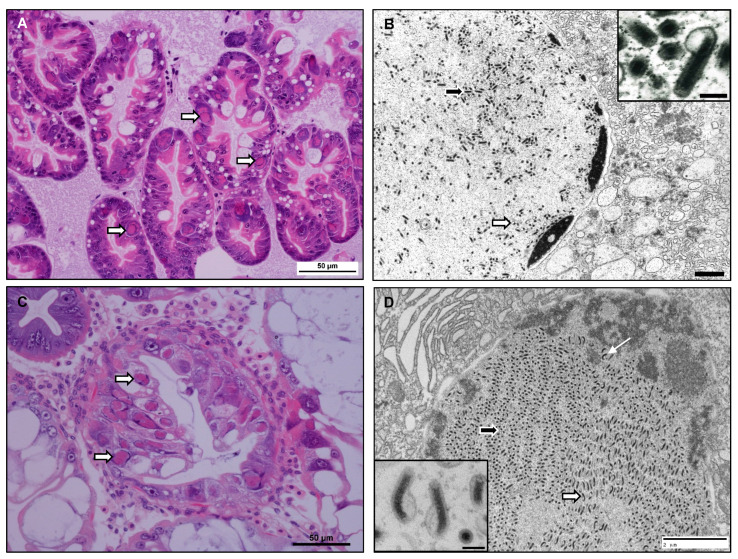
(**A**) Crangon crangon Nudivirus (CcNV) infected nuclei within hepatopancreas tubules. Infected nuclei (arrows) are enlarged with marginalised chromatin and contain an eosinophilic inclusion body. H&E stain. Scale bar = 50 µm. (**B**) CcNV virions within an infected nucleus. Virions contain a rod shaped, electron dense nucleocapsid surrounded by a trilaminar envelope (inset, scale bar = 100 nm). Virions have been caught in cross section (black arrow) and longitudinal section (white arrow) within the nucleus. TEM. Scale bar = 1 µm. (**C**) Carcinus maenas Nudivirus (CmNV) infected nuclei within hepatopancreas tubules. Infected nuclei (arrows) are enlarged with marginalised chromatin and contain an eosinophilic inclusion body. H&E stain. Scale bar = 50 µm. (**D**) CmNV virions within an infected nucleus. Virions contain a rod shaped, electron dense nucleocapsid surrounded by a trilaminar envelope. Virions have been caught in cross section (black arrow) and longitudinal section (white arrow) within the nucleus. Some of the nucleocapsids appear curved (inset, scale bar = 100 nm) or u shaped (line arrow) within the envelope. TEM. Scale bar = 2 µm.

A total of 50 *C. crangon* were sampled, 90% of which were shown to be positive for infection with a bacilliform virus via histological analysis (Figure 1). Infected nuclei within the hepatopancreatic epithelial cells were hypertrophied with marginalised chromatin and contained eosinophilic inclusion bodies (Figure 1A). By applying a previously established severity grading scheme [23] for the pathology level of the infections, we revealed that 10% of shrimp were Grade 0 (uninfected), 20% Grade 1, 20% Grade 2, 30% Grade 3 and 20% Grade 4 infected. TEM showed enveloped virions with rod-shaped nucleocapsids and with a characteristic expansion of the envelope at one end (Figure 1B), as previously described for the bacilliform virus [21].

As described by Bojko et al. [24], a bacilliform virus was also identified in 17.4% of *C. maenas* crabs sampled from Canada (*n* = 432). Pathology was similar to that described by Stentiford and Feist [23], with hepatopancreatic epithelial cells showing enlarged nuclei with marginalised chromatin and eosinophilic inclusion bodies (Figure 1C). Using the severity grading scheme [23] the majority of infections were shown to be Grade 1 (76%, 20% Grade 2, 2% Grade 3 and 2% Grade 4). TEM revealed rod-shaped nucleocapsids within an envelope and with an extension at one end, however, unlike the virus in *C. crangon*, some of these nucleocapsids appeared as slightly bent and u-shaped within the envelopes (Figure 1D). The crab-derived virions were also slightly larger measuring 340 nm by 75 nm (*n* = 30), compared to the CcNV virions which measured 280 nm by 71.8 nm [21].

### 3.2. De Novo Genome Assembly of Two Novel Nudiviruses

Illumina sequencing resulted in the generation of 843,215 and 2,601,878 read pairs for the *Crangon crangon* and *Carcinus maenas* samples, respectively. The number of read pairs remaining for the shrimp and crab samples after quality-trimming were 837,743 (99.4%) and 1,720,539 (66.1%) read pairs, respectively, and after normalisation, 175,562 and 250,312 read pairs, respectively. A total of 613,455 Nanopore reads were obtained with lengths ranging from 62 bp to 46,624 nt and a mean length of 748 nt. After removal of barcode sequences and reads <1000 nt in size, 66,775 reads remained with a mean length of 2184 nt.

**Figure 2:**
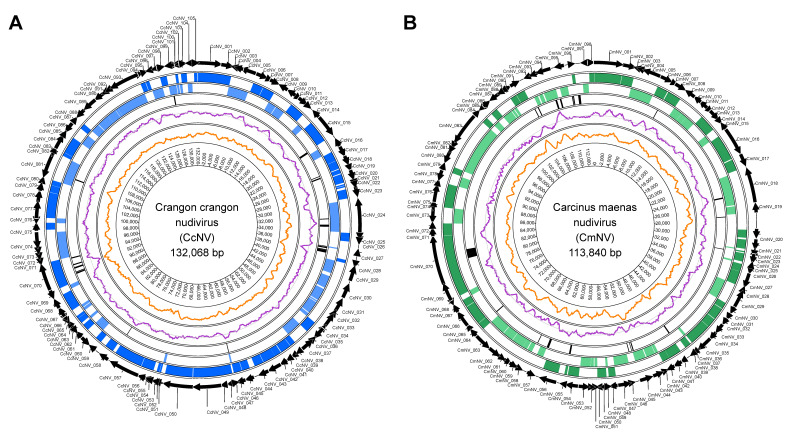
Circular genomic maps of (**A**) Crangon crangon nudivirus (CcNV) and (**B**) Carcinus maenas nudivirus (CmNV). The outermost to innermost tracks represent: (1) predicted protein-coding genes and their orientation, (2) predicted protein-coding genes on the forward strand, (3) protein-coding genes on the reverse strand, (4) tandem repeat regions, (5) GC content and (6) GC skew, (7) genome coordinates.

Assembly of the *Crangon crangon* quality-trimmed and normalised Illumina sequences alone resulted in the generation of four contigs, of which the largest two contigs (89,711 nt and 41,818 nt in size) were shown to represent novel nudivirus genome sequences by blastn similarity searches. The third contig (11,337 nt) represented *Crangon crangon* 28S and 18S ribosomal DNA sequences, whereas the fourth contig (291 nt) did not have any hits with any of the sequences in the NCBI database. A hybrid assembly using both the Illumina read sequences and the Nanopore sequences produced two contigs, the largest of which represented a circular putative CcNV genome sequence of 132,068 nt in length, with a read coverage of 338x and a GC content of 29.5% (Figure 2A). The second contig (11,337 nt) was identical to the third contig generated by the Illumina sequences assembly only and represented *Crangon crangon* 28S/18S ribosomal DNA sequences. De novo assembly of the *C. maenas* sample using the normalised quality-trimmed Illumina reads resulted in 27 assembled contig sequences, the largest of which was a circular sequence of 113,840 nt in length, with a read coverage of 995× and a GC content of 38.8% (Figure 2B). Using blastn similarity searches, this sequence also represented a novel putative nudivirus genome sequence; the other sequences showed high similarity to 18S ribosomal RNA, mitochondrial and microsatellite sequences of the host.

### 3.3. Characterisation of the Nudivirus Genomes

#### 3.3.1. Open Reading Frame (ORF) Prediction

Annotation of the genome sequences identified 106 (ranging from 51 to 1488 nt) and 99 (ranging from 41 to 1828 nt in length) predicted protein sequences for CcNV and CmNV, respectively (based on a consensus prediction of open reading frames (ORFs) by four different software tools) as shown in Table 1 and Table 2, and Tables S1 and S2 in the Supplemental File S1. The gene densities for CcNV and CmNV were 1.3 and 1.2 genes per kb, respectively. 

#### 3.3.2. Tandem Repeats

A total of 36 tandem repeats covering 19 separate regions were identified in the CcNV genome, ten of which overlapped predicted ORFs (Table S3). The lengths of these regions ranged from 58 to 367 nt, covering 3057 nt or 2.3% of the genome. In the CmNV genome, 39 tandem repeats were found that covered 22 genome regions. These regions ranged from 56 to 552 nt in length, with a total length of 3838 nt (3.4% of the genome) and overlapped with eight predicted ORFs (Table S4).

#### 3.3.3. Protein Orthology and Gene Content

OrthoFinder assigned 1265 of the 1786 nudivirus and Autographa californica multiple nucleopolyhedrovirus genes (71.0% of total) to 237 orthogroups. In total, 50% of all genes were in orthogroups with five or more genes (G50 = 5) and were contained in the largest 103 orthogroups (O50 = 103). There were seven orthogroups with all species present and six of these consisted entirely of single-copy genes. The orthogroups and the single copy orthologues are listed in Table S5 in the Supplemental Material, along with gene descriptions derived from the corresponding NCBI nucleotide records. 

We were not able to find an ortholog of the baculovirus major capsid protein VP39 in CcNV and CmNV, but we did find a homolog of the major capsid protein identified through proteomic analysis in ToNV (ToNV ORF87; CcNV ORF14 and CmNV ORF15) [8]. This protein was found in 4 different orthogroups: OG0000071 (which includes DiNV ORF99, ENV ORF57, GbNV ORF64, KNV ORF69, MNV ORF63, OrNV ORF15 and TNV ORF18), OG0000729 (ToNV ORF 87), OG0000204 (HzNV-1 ORF89 and HzNV-2 ORF52 and OG0000098 (CcNV ORF14, CmNV ORF15, DhNV ORF14, HgNV ORF15 and PmNV ORF22 (Table S5). Reciprocal BLAST searches confirmed that the VP39 protein sequences of the nudiviruses in OG0000071 were similar, but different from the proteins in the other orthogroups. The protein sequences across OG0000729, OG0000204, OG0000098 were also found to be similar using BLAST searches. 

Using orthology analysis and reciprocal BLAST searches we could not identify p6.9 in the CcNV and CmNV proteomes. It has been shown that the p6.9 homolog of OrNV and related alphanudiviruses from drosophilid hosts is a fusion of the homologs of GbNV p6.9 (ORF73) and GbNV ORF72 [4]. In other nudiviruses sequenced to date, this gene has been identified as an independent ORF; however, for some nudiviruses including DhNV, HzNV-1, HzNV-2 and PmNV this gene was not annotated in NCBI. The most likely explanation for this could be the repetitive serine (S) and arginine (R) sequences in the deduced protein sequences, which may not be identified by gene prediction tools as being part of potential protein sequences. Bezier et al. [8] identified separate ORFs for p6.9 in HzNV-1 (ORF142; position 210,245–210,493), HzNV-2 (position 24,375–24,127) and PmNV (position 64,881–65,078) and in the current study, this gene was identified in CcNV (72,007–72,231) and CmNV (position 45,460–45,651) using custom BLAST searches. However, a p6.9 homolog could not be found in the DhNV genome.

Another protein previously considered as a core gene, LEF-5, was found in the proteomes of all nudiviruses, apart from ENV, KNV and MNV. Using the vgas gene prediction tool [39] that was used for CcNV and CmNV in this study, we identified this gene in the genomes of all three nudiviruses: ENV (position 74,295–74,462), KNV (position 120,410–120,246) and MNV (position 57,038–57,274). Furthermore, GbNV_51-like was present in the proteomes of all nudiviruses apart from HzNV-1. However, in a previous study, this gene was found to be located between ORF57 and ORF 58 (position 79,463 to 79,900) in the HzNV-1 genome [8]. Previous work has also shown that the genes encoding for P47 and LEF-9 homologs are fused in the HzNV-1 and HzNV-2 genomes [8,18].

**Figure 3:**
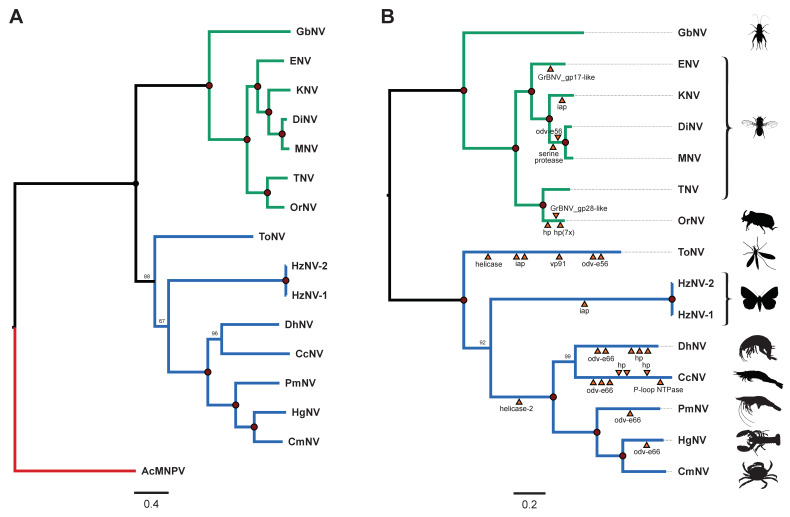
Phylogenetic trees based on concatenated nudivirus protein sequences. (**A**)—Maximum likelihood phylogeny based on a supermatrix of 19 concatenated pruned orthologous protein sequences (6961 amino acids; 38K protein, Ac81-like protein, DNA polymerase, FEN-1, GbNV_gp67-like, integrase, LEF-4, LEF-8, LEF-9, P33, PIF-0, PIF-1, PIF-2, PIF-3, PIF-4, PIF-6, TK3, VLF-1 and VP91) derived from 15 nudiviruses and Autographa californica multiple nucleopolyhedrovirus (AcMNPV; NC_001623.1), which was used as the outgroup. (**B**). Maximum likelihood phylogeny based on 27 concatenated nudivirus core genes (5244 amino acids in total) of 15 nudiviruses. Gene duplication events identified by OrthoFinder are indicated by orange triangles. hp = hypothetical protein. Node labels indicate bootstrap support expressed as a percentage and solid red dots indicate 100% support. CcNV = Crangon crangon nudivirus (MZ311577), CmNV = Carcinus maenas nudivirus (MZ311578), DhNV = Dikerogammarus haemobaphes nudivirus (MT488302.1), DiNV = Drosophila innubila nudivirus (NC_040699.1), ENV = Esparto virus (NC_040536.1), GbNV = Gryllus bimaculatus nudivirus (NC_009240.1), HgNV = Homarus gammarus nudivirus (MK439999.1), HzNV-1 = Heliothis nudivirus 1 (AF451898.1), HzNV-2 = Heliothis nudivirus 2 (NC_004156.2), KNV = Kallithea virus (NC_033829.1), MNV = Mauternbach virus (MG969167), OrNV = Oryctes rhinoceros nudivirus (NC_011588.1), PmNV = Penaeus monodon nudivirus (KJ184318), TNV = Tomelloso virus (NC_040789.1), ToNV = Tipula oleracea nudivirus (NC_026242.1).

Remarkable was also the absence of the three thymidine kinase genes in CcNV, while in GmNV and in all other sequenced nudivirus genomes, three genes for thymidine synthesis have been identified (*tk1-3*). Compared to most other nudiviruses found in non-aquatic host species, CcNV has additional copies of the gene encoding orthologues for the baculovirus ODV-E66 protein (a total of five copies). Orthology analysis indicated that CcNV ORF29 originated from an ancestral gene shared by all other nudiviruses that contain *odv-e66* in their genome and that it was duplicated three times, resulting in ORFs 30, 31 and 33 (Figure 3B). An additional ODV-E66 homolog (CcNV ORF23) appears to be acquired by CcNV independently. All ODV-E66 genes are in close vicinity to each other in the genome and the similarity between these copies ranges between 42 and 85 percent at the nucleotide level. Similarly, gene duplication events resulted in multiple copies of *odv-e66* in the genomes of DhNV (ORF18, ORF21 and ORF23), PmNV (ORF34 and ORF36) and HgNV (ORF24 and ORF25). Interestingly, only a single copy of this gene was found in CmNV (ORF56; Figure 3B). The baculovirus ODV-E66 protein plays an important role in oral infectivity and may help the virus to overcome the peritrophic membrane lining the midgut [50]. Whether increased levels of ODV-E66 would give these two nudiviruses a particular benefit in their crustacean hosts or their environment awaits further analysis.

#### 3.3.4. Phylogenetic Analysis Revealed Two Mayor Lineages within the Nudiviruses

After removal of paralogues and gene trees with less than 12 species using PhyloTreePruner, 19 pruned orthogroups remained. Phylogenetic analyses were conducted using a supermatrix of the 19 concatenated pruned orthologous protein sequences (6961 amino acids in total), which included: 38K protein, Ac81-like protein, DNA polymerase, FEN-1, GbNV_gp67-like, integrase, LEF-4, LEF-8, LEF-9, P33, PIF-0, PIF-1, PIF-2, PIF-3, PIF-4, PIF-6, TK3, VLF-1 and VP91. In this phylogeny we used the baculovirus AcMNPV as an outgroup. Maximum Likelihood analyses placed CmNV together with PmNV and HgNV. CcNV was placed with DhNV into a separate clade (with bootstrap support values of 100). Both of these clades were part of a larger clade that also contained HzNV-1 and HzNV-2, and ToNV with bootstrap support values of 67 and 88, respectively (Figure 3A).

To further analyse the evolutionary relationship between the various nudiviruses, we conducted phylogenetic analysis as described above, but this time based on a supermatrix of the deduced amino acid sequences of all genes shared by nudiviruses (5244 amino acids; the p6.9 protein sequence was excluded for these analyses as no sequence was (yet) obtained for DhNV), the nudiviruses core genes (Figure 3B). This analysis further emphasized the demarcation of two major lineages within the nudiviruses as a whole.

#### 3.3.5. Nudivirus Core Gene Analysis

**Figure 4:**
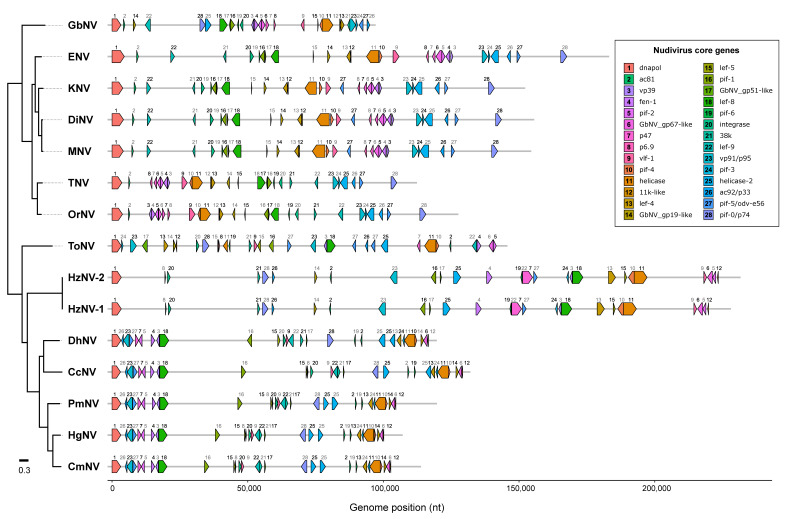
Genome map of 15 nudiviruses showing the position of 28 core genes. Open reading frames (ORFs) are shown as arrows, which indicate the gene position and orientation in the genome. Grey lines represent the full length (nt) of the genomes. The genome sequences were rearranged, such that all linear representations of the circular viral genomes start with the DNA polymerase gene. The genome map was created using the R package gggenes and combined with the phylogenetic tree shown in Figure 3B using Inkscape.

Based on orthology analysis and reciprocal blast searches, a total of 28 nudivirus core genes were identified across the 15 nudiviruses (see Table 3). As highlighted in Figure 4, the nudiviruses described from aquatic hosts (DhNV, CcNV, PmNV, HgNV and CmNV) show highly similar gene order and orientation within the genome when compared with terrestrial hosts. Viral genomes described from hosts in marine environments (CcNV, PmNV, HgNV and CmNV) showed identical gene order within the genomes, slight variation in gene order and orientation was shown in DhNV which is described in a freshwater host (Figure 4). 

## 4. Discussion

We used genomic data to characterise two previously described viruses infecting crustaceans, Crangon crangon bacilliform virus and Carcinus maenas bacilliform virus. Histological and ultrastructural details highlight morphological similarities between these viruses and viruses described from the hepatopancreas of other crustacean species (*Penaeus monodon*, *Homarus gammarus* and *Dikerogammerus haemobaphes*). Infected nuclei within the hepatopancreatic epithelial cells appeared hypertrophied with marginalised chromatin and contained eosinophilic inclusion bodies. Although appearing similar histologically, electron microscopy identified differences in the appearance of the virions between the two infections. Virions in *C. crangon* appeared smaller in size with straight nucleocapsids within the envelope, virions in *C. maenas* were larger and possessed a curved, slightly bent nucleocapsid within the envelope. Severity of infection also appeared to show variation with a larger number of *C. crangon* samples shown to be infected with Grade 3 and 4 infections when compared to *C. maenas*.

There are, however, currently no cell lines available for crustacean tissues [51], meaning that many classical viral classification techniques are not available to characterise crustacean viruses. The ability to generate full-length DNA genomes, as opposed to a set of separate contig sequences of novel pathogens), however significantly improves the classification and characterisation of crustacean DNA viruses. We have developed protocols to enable sequencing of large, non-culturable viral DNA genomes, involving viral DNA purification steps, sequencing on an Illumina MiSeq, and subsequent analysis of sequence data. One of the intrinsic limitations of Illumina sequencing, is that it is not capable of sequencing (large) repeat regions and regions of low complexity due to read length limitations, which in our case also prevented the assembly of full-length genome sequences. The additional use of MinIon technology provided long read sections of the genomes, allowing us to determine the order of the available contigs and to obtain complete, circular genomes. We used the complete genomes to study their evolutionary relationship and their position within described viral families.

The genome size, number of ORFs and presence of conserved genes in the two novel virus sequences shows that the two crustacean dsDNA viruses belong to the family *Nudiviridae*. Utilizing the new latinized binominal method for the naming of virus species, we propose they are named *Gammanudivirus* c*racrangoni* and *Gammanudivirus camaenasi*, with the common names Crangon crangon nudivirus (CcNV) and Carcinus maenas nudivirus (CmNV) respectively. Their position in phylogenetic trees (Figure 3) indicated that these viruses each belong to separate clades and are genetically distinct from other viruses within these clades, thus should be classified as distinct species.

When comparing the gene content, it became clear that CcNV lacked the three thymidine kinase genes (*tk1-3*), found thus far in all other nudiviruses. Instead of encoding its own enzymes to synthesize thymidine monophosphate, a crucial molecule in DNA synthesis and viral DNA replication, CcNV apparently relies on the host’s machinery for this process. A similar situation is seen in baculoviruses that also do not have any *tk* genes. Whether the absence of these genes affects the fitness of this virus is not clear, but from the histological images presented here (Figure 1) and reported in other studies [21], it appears that CcNV replicates to high levels, despite the absence of viral *tk* genes.

Homologs for p6.9 were found in all nudiviruses, with the exception for DhNV. This gene encodes a small arginine- and serine-rich protein and plays an essential role in various viral physiological processes during infection [52,53]. It is conserved across all baculoviruses and nudiviruses and it is highly likely that this gene is also present in DhNV and that it has yet to be discovered.

The presence or absence of an obvious ortholog of the baculovirus major capsid protein VP39 (or evolutionary related protein) has not been identified with certainty for all nudiviruses. *Alphanudiviruses* and *Betanudiviruses* all have such an ortholog. In contrast, viruses in the genera *Deltanudivirus* and *Gammanudivirus* appeared to have a functional homolog in the form of a capsid protein of approximately 34 kDa, as first identified in ToNV (ORF087) through proteomic analysis [8]. Based on our analyses, we consider CcNV ORF14, CmNV ORF15, DhNV ORF14, HgNV ORF15, HzNV-1 ORF89, HzNV-2 ORF52, PmNV ORF22 and ToNV ORF87 to be vp39 homologs. We have therefore found VP39 homologs in all described nudiviruses and have included this gene within the core gene list, bringing the total number of nudivirus core genes to 28, as presented here. Interestingly, we show that nudiviruses described from aquatic hosts (DhNV, CcNV, PmNV, HgNV and CmNV) possess a highly similar gene order and orientation within the genome when compared with nudiviruses from terrestrial hosts. Viral genomes described from hosts in marine environments (CcNV, PmNV, HgNV and CmNV) showing identical gene order and orientation within the genomes (Figure 4). The genome of DhNV, described in a freshwater host, displayed variation in the orientation of the pif-0/p74 gene and in the gene order and orientation of the pif-6, ac81 and helicase 2 genes when compared with viral genomes from hosts in the marine environment. As highlighted previously, we expect further nudivirus infections affecting aquatic crustaceans to be identified from both marine and freshwater systems. Further comparisons between viral genomes from the two environments, in particular gene order and orientation, will be possible as these are described.

Within the family *Nudiviridae*, the ICTV has recently approved a proposal for four parallel genera [4]. A subdivision of the family into five parallel genera (*Alphanudivirus* to *Epsilonnudivirus*) has also been proposed [8,12,19], however, after studying core gene content and comparing the data between all described nudivirus species, we believe this subdivision in five parallel genera is no longer supported by the data. After the addition of the two aquatic nudiviruses (CmNV and CcNV, (Figure 3)) described in this paper, we observe a clear demarcation of two major clades) in the nudivirus phylogeny. The first clade contains nudiviruses found in *Drosophila* (Diptera: suborder Brachycera), Coleoptera and Orthoptera, while the second clade harbours nudiviruses isolated from crustaceans, *Heliothis zea* moths (Lepidoptera) and the marsh crane fly *Tipula oleracea* (Diptera: suborder Nimatocera). Within these two clades, subclades begin to appear that may be the basis for distinct taxonomic genera. We therefore propose to create two subfamilies within the family *Nudiviridae*, and sub-divide these further into genera. Recently, Liu et al. [9] came to a similar conclusion after analysing nudiviruses from two corn rootworm species (order Coleoptera) and gave a suggestion for the naming of the respective taxons. The subfamilies would be called *Alphanudivirinae* and *Betanudivirinae,* and the new CcNV and CmNV nudiviruses would group together with the other crustacean nudiviruses. However, after adding CcNV and CmNV sequences the phylogeny no longer shows a clear demarcation between the species placed by Liu et al. [9] in a proposed genus *Helnudivirus* (HzNV1-2 and ToNV) and the crustacean infecting nudiviruses, for which the genus name *Malnudivirus* was proposed (derived from the term class *Malacostraca).* Based on our data, a further subdivision of the subfamily *Betanudivirinae* is therefore not currently warranted. 

## 5. Conclusions

In conclusion, we have shown, using histological, ultrastructural and genomic data that the bacilliform viruses infecting the two aquatic crustacean species, *C. crangon* and *C. maenas,* should be classified in separate genera of the family *Nudiviridae* under the species *Gammanudivirus* c*racrangoni* (Crangon crangon nudivirus) and *Gammanudivirus camaenasi* (Carcinus maenas nudivirus). The compiled genomic and phylogenetic information presented here provide further support for an alternative structure of the nudivirus family, as recently also proposed by Liu et al. [9], in which the *Nudiviridae* family will harbour the two subfamilies *Alphanudivirinae* and *Betanudivirinae*, each of which is subdivided into several genera.

## Figures and Tables

**Table 1:** Identified open reading frames (ORFs) in the CcNV genome. The start and end positions of the ORFs are shown, as well as the strand it was found on, the deduced protein length (in amino acids), the best hits obtained using blastp similarity searches using the NCBI nr protein database (E-value < 0.001), and additional information obtained from annotated orthogroups (Table S5). Nudivirus core genes are highlighted in grey.

Gene_ID (ORF)	Start	End	Strand	Protein Length (Amino Acids)	Best Hit (Description, Species, Accession Number)	Additional Annotations
CcNV_1	1	3111	+	1037	DNA polymerase (Dikerogammarus haemobaphes nudivirus) (QLI62362.1)	
CcNV_2	3114	4613	+	500	hypothetical protein (Apolygus lucorum) (KAF6208213.1)	Methyltransferase
CcNV_3	4617	4874	-	86	KN57_gp007 (Dikerogammarus haemobaphes nudivirus) (QLI62363.1)	
CcNV_4	4877	5572	-	232	Ac92 (Dikerogammarus haemobaphes nudivirus) (QLI62364.1)	P33
CcNV_5	5544	7580	-	679	Vp91 (Penaeus monodon nudivirus) (YP_009051847.1)	
CcNV_6	7843	9279	+	479	ODV-E56 (Dikerogammarus haemobaphes nudivirus) (QLI62366.1)	PIF-5
CcNV_7	9357	9833	+	159	KN57_gp012 (Dikerogammarus haemobaphes nudivirus) (QLI62367.1)	
CcNV_8	9802	10995	+	398	p47 (Dikerogammarus haemobaphes nudivirus) (QLI62368.1)	
CcNV_9	11,007	12,209	-	401	Pif-2 (Homarus gammarus nudivirus) (YP_010087649.1)	
CcNV_10	12,317	13,708	-	464	-	
CcNV_11	13,882	14,199	+	106	-	
CcNV_12	14,208	15,503	+	432	KN57_gp020 (Dikerogammarus haemobaphes nudivirus) (QLI62373.1)	FEN-1
CcNV_13	15,500	15,985	+	162	HgNV_014 (Dikerogammarus haemobaphes nudivirus) (QLI62374.1)	
CcNV_14	16,535	17,410	-	292	hypothetical protein PmNV_022 (Penaeus monodon nudivirus) (YP_009051860.1)	VP39
CcNV_15	17,589	20,660	+	1024	LEF-8 (Dikerogammarus haemobaphes nudivirus) (QLI62376.1)	
CcNV_16	21,228	22,379	-	384	-	
CcNV_17	22,523	23,827	+	435	-	
CcNV_18	23,881	25,026	+	382	-	
CcNV_19	25,154	25,723	+	190	-	
CcNV_20	25,863	27,080	+	406	p51 (Dikerogammarus haemobaphes nudivirus) (QLI62377.1)	
CcNV_21	27,305	27,478	+	58	-	
CcNV_22	27,552	27,737	+	62	-	
CcNV_23	28,064	29,332	+	423	ODV-E66 (Penaeus monodon nudivirus) (YP_009051872.1)	
CcNV_24	29,595	34,061	-	1489	DhNV_024 (Dikerogammarus haemobaphes nudivirus) (QLI62385.1)	
CcNV_25	34,158	34,835	+	226	-	
CcNV_26	34,858	35,337	+	160	-	dUTP pyrophosphatase/dUTPase
CcNV_27	36,459	36,614	-	52	-	
CcNV_28	37,945	38,391	+	149	-	
CcNV_29	38,420	40,342	-	641	ODV_E66 (Dikerogammarus haemobaphes nudivirus) (QLI62384.1)	
CcNV_30	40,871	42,697	-	609	ODV_E66 (Dikerogammarus haemobaphes nudivirus) (QLI62384.1)	
CcNV_31	42,958	44,778	-	607	ODV_E66 (Dikerogammarus haemobaphes nudivirus) (QLI62384.1)	
CcNV_32	44,932	45,108	-	59	-	
CcNV_33	45,218	47,050	-	611	ODV_E66 (Dikerogammarus haemobaphes nudivirus) (QLI62384.1)	
CcNV_34	47,201	47,683	-	161	-	
CcNV_35	47,675	49,270	+	532	PIF-1 (Penaeus monodon nudivirus) (YP_009051877.1)	
CcNV_36	49,285	49,566	-	94	-	
CcNV_37	49,556	51,547	-	664	HgNV_030 (Dikerogammarus haemobaphes nudivirus) (QLI62398.1)	
CcNV_38	51,546	52,316	+	257	DhNV_036 (Dikerogammarus haemobaphes nudivirus) (QLI62397.1)	
CcNV_39	52,382	53,083	+	234	-	
CcNV_40	53,107	53,847	+	247	HgNV_033 (Dikerogammarus haemobaphes nudivirus) (QLI62395.1)	PmV-like protein
CcNV_41	53,962	54,891	+	310	hypothetical protein (Penaeus monodon nucleopolyhedrovirus) (ABX44696.1)	
CcNV_42	54,922	56,667	+	582	KN57_gp048 (Dikerogammarus haemobaphes nudivirus) (QLI62392.1)	
CcNV_43	56,737	58,020	+	428	-	
CcNV_44	58,774	59,850	+	359	-	
CcNV_45	59,923	61,056	+	378	-	
CcNV_46	60,983	61,204	-	74	-	
CcNV_47	61,297	62,304	+	336	-	
CcNV_48	62,323	63,135	+	271	-	Polyhedrin
CcNV_49	63,152	66,742	+	1197	-	
CcNV_50	66,844	70,668	+	1275	-	
CcNV_51	70,945	71,277	-	111	-	
CcNV_52	71,252	71,461	+	70	Ac92 (Dikerogammarus haemobaphes nudivirus) (QLI62411.1)	
CcNV_53	71,517	71,873	+	119	-	LEF-5
CcNV_54	72,349	72,531	+	61	hypothetical protein PmNV_053 (Penaeus monodon nudivirus) (YP_009051891.1)	
CcNV_55	72,528	73,169	+	214	hypothetical protein, partial (Penaeus monodon nucleopolyhedrovirus) (ABX44701.1)	
CcNV_56	73,166	74,092	+	309	Integrase (Dikerogammarus haemobaphes nudivirus) (QLI62415.1)	
CcNV_57	74,169	78,131	+	1321	hypothetical protein KM727_gp62 (Homarus gammarus nudivirus) (YP_010087702.1)	
CcNV_58	78,592	79,830	+	413	non-structural protein 1 (Penaeus monodon metallodensovirus) (QGX07563.1)	
CcNV_59	80,670	81,551	+	294	VLF-1 (Dikerogammarus haemobaphes nudivirus) (QLI62416.1)	
CcNV_60	81,546	83,069	-	508	LEF-9 (Dikerogammarus haemobaphes nudivirus) (QLI62417.1)	
CcNV_61	83,137	84,054	+	306	38K protein (Penaeus monodon nudivirus) (YP_009051897.1)	
CcNV_62	84,031	84,408	-	126	-	
CcNV_63	84,420	85,166	+	249	HgNV_049 (Dikerogammarus haemobaphes nudivirus) (QLI62423.1)	
CcNV_64	85,212	85,556	+	115	-	GbNV_gp51-like
CcNV_65	85,560	85,988	-	143	-	
CcNV_66	86,110	86,490	-	127	-	
CcNV_67	86,589	87,866	+	426	KN57gp_066 (Dikerogammarus haemobaphes nudivirus) (QLI62428.1)	
CcNV_68	87,891	89,111	+	407	-	
CcNV_69	89,326	89,904	-	193	-	
CcNV_70	90,070	93,882	+	1271	-	Polyhedrin
CcNV_71	93,921	94,484	-	188	KN57gp_068 (Dikerogammarus haemobaphes nudivirus) (QLI62436.1)	
CcNV_72	94,498	95,367	-	290	KN57gp_069 (Dikerogammarus haemobaphes nudivirus) (QLI62435.1)	
CcNV_73	95,355	95,990	-	212	KN57gp_070 (Dikerogammarus haemobaphes nudivirus) (QLI62434.1)	
CcNV_74	95,996	97,999	-	668	P74 (Homarus gammarus nudivirus) (YP_010087703.1)	PIF-0
CcNV_75	98,378	99,340	+	321	-	
CcNV_76	99,315	100,034	-	240	HgNV_068 (Dikerogammarus haemobaphes nudivirus) (QLI62449.1)	
CcNV_77	100,025	102,127	+	701	Helicase 2 (Dikerogammarus haemobaphes nudivirus) (QLI62448.1)	
CcNV_78	102,130	103,560	+	477	-	
CcNV_79	103,617	104,579	+	321	DhNV_085 (Dikerogammarus haemobaphes nudivirus) (QLI62446.1)	
CcNV_80	104,623	105,300	+	226	-	
CcNV_81	105,322	108,495	-	1058	-	
CcNV_82	108,501	108,920	-	140	HgNV_075 (Dikerogammarus haemobaphes nudivirus) (QLI62443.1)	
CcNV_83	108,991	109,458	+	156	Ac81 (Dikerogammarus haemobaphes nudivirus) (QLI62442.1)	
CcNV_84	109,506	111,398	+	631	hypothetical protein PmNV_087 (Penaeus monodon nudivirus) (YP_009051925.1)	
CcNV_85	111,385	111,831	+	149	Ac68-like protein (Homarus gammarus nudivirus) (YP_010087718.1)	PIF-6
CcNV_86	111,842	113,356	-	505	DhNV_068 (Dikerogammarus haemobaphes nudivirus) (QLI62429.1)	
CcNV_87	113,499	114,260	+	254	VLF-1 (Dikerogammarus haemobaphes nudivirus) (QLI62430.1)	
CcNV_88	114,264	115,541	-	426	-	
CcNV_89	115,658	117,484	-	609	Helicase 2 (Dikerogammarus haemobaphes nudivirus) (QLI62451.1)	
CcNV_90	117,522	118,901	-	460	LEF-4 (Dikerogammarus haemobaphes nudivirus) (QLI62453.1)	
CcNV_91	118,976	119,311	-	112	-	
CcNV_92	119,355	120,035	-	227	PIF-3 (Homarus gammarus nudivirus) (YP_010087723.1)	
CcNV_93	120,035	123,916	-	1294	Helicase (Dikerogammarus haemobaphes nudivirus) (QLI62456.1)	
CcNV_94	123,918	124,592	+	225	ODV-E28 (Dikerogammarus haemobaphes nudivirus) (QLI62457.1)	PIF-4
CcNV_95	124,681	125,151	+	157	-	
CcNV_96	125,168	125,998	-	277	-	
CcNV_97	126,024	126,755	-	244	KN57gp_097 (Dikerogammarus haemobaphes nudivirus) (QLI62459.1)	
CcNV_98	126,754	127,662	+	303	Esterase (Dikerogammarus haemobaphes nudivirus) (QLI62460.1)	GbNV_gp19-like
CcNV_99	127,679	128,800	-	374	KN57gp_099 (Dikerogammarus haemobaphes nudivirus) (QLI62461.1)	GbNV_gp67-like
CcNV_100	128,897	129,250	+	118	-	
CcNV_101	128,905	129,216	-	104	11K (Dikerogammarus haemobaphes nudivirus) (QLI62462.1)	
CcNV_102	129,348	129,707	-	120	-	
CcNV_103	129,688	130,482	+	265	KN57gp_102 (Dikerogammarus haemobaphes nudivirus) (QLI62464.1)	
CcNV_104	130,530	131,210	-	227	KN57gp_107 (Dikerogammarus haemobaphes nudivirus) (QLI62465.1)	
CcNV_105	131,513	131,980	+	156	-	
CcNV_106	72,007	72,231	+	75	-	p6.9

**Table 2:** Identified open reading frames (ORFs) in the CmNV genome. The start and end positions of the ORFs are shown, as well as the strand it was found on, the deduced protein length (in amino acids), the best hits obtained using blastp similarity searches using the NCBI nr protein database (E-value < 0.001), and additional information obtained from annotated orthogroups (Table S5). Nudivirus core genes are highlighted in grey.

Gene_ID (ORF)	Start	End	Strand	Protein Length (Amino Acids)	Best Hit (Description, Species, Accession Number)	Additional Annotations
CmNV_1	1	3200	+	1066	DNA polymerase (Homarus gammarus nudivirus) (YP_010087641.1)	
CmNV_2	3264	4663	+	466	methyltransferase (Homarus gammarus nudivirus) (YP_010087642.1)	
CmNV_3	4669	4880	-	70	hypothetical protein PmNV_007 (Penaeus monodon nudivirus) (YP_009051845.1)	
CmNV_4	4868	5535	-	222	Ac92-like protein (Homarus gammarus nudivirus) (YP_010087644.1)	P33
CmNV_5	5523	7579	-	685	Vp91 (Homarus gammarus nudivirus) (YP_010087645.1)	
CmNV_6	7716	9013	+	432	ODV-E56 (Homarus gammarus nudivirus) (YP_010087646.1)	PIF-5
CmNV_7	9132	9526	+	131	hypothetical protein KM727_gp07 (Homarus gammarus nudivirus) (YP_010087647.1)	
CmNV_8	9537	10,774	+	412	P47 (Homarus gammarus nudivirus) (YP_010087648.1)	
CmNV_9	10,793	11,949	-	385	Pif-2 (Homarus gammarus nudivirus) (YP_010087649.1)	
CmNV_10	11,991	12,712	-	240	HZV 115-like protein (Homarus gammarus nudivirus) (YP_010087650.1)	
CmNV_11	12,750	13,996	-	415	hypothetical protein KM727_gp11 (Homarus gammarus nudivirus) (YP_010087651.1)	
CmNV_12	14,119	14,495	+	125	hypothetical protein KM727_gp12 (Homarus gammarus nudivirus) (YP_010087652.1)	
CmNV_13	14,469	15,715	+	415	hypothetical protein KM727_gp13 (Homarus gammarus nudivirus) (YP_010087653.1)	FEN-1
CmNV_14	15,706	16,145	+	146	hypothetical protein KM727_gp14 (Homarus gammarus nudivirus) (YP_010087654.1)	
CmNV_15	16,174	17,063	-	296	Vp39/31 k (Homarus gammarus nudivirus) (YP_010087655.1)	VP39
CmNV_16	17,183	20,256	+	1024	LEF-8 (Homarus gammarus nudivirus) (YP_010087656.1)	
CmNV_17	20,397	21,742	+	448	P51 (Homarus gammarus nudivirus) (YP_010087657.1)	
CmNV_18	21,993	25,855	-	1287	hypothetical protein KM727_gp18 (Homarus gammarus nudivirus) (YP_010087658.1)	
CmNV_19	26,020	27,005	-	328		E3 ubiquitin-protein ligase TRIM39-like protein
CmNV_20	29,001	29,953	+	317		
CmNV_21	30,175	31,274	+	366	serine/threonine protein kinase (Homarus gammarus nudivirus) (YP_010087663.1)	
CmNV_22	31,337	31,461	-	41		
CmNV_23	31,567	31,706	+	46		
CmNV_24	31,838	32,829	+	330	dihydroxy-acid dehydratase (Homarus gammarus nudivirus) (YP_010087666.1)	
CmNV_25	32,866	33,068	-	67		
CmNV_26	33,141	34,036	+	298	guanosine monophosphate kinase (Homarus gammarus nudivirus) (YP_010087667.1)	TK2
CmNV_27	34,073	35,664	+	530	PIF-1 (Homarus gammarus nudivirus) (YP_010087668.1)	
CmNV_28	35,695	36,065	-	123	hypothetical protein PmNV_040 (Penaeus monodon nudivirus) (YP_009051878.1)	
CmNV_29	36,056	38,163	-	702	hypothetical protein KM727_gp30 (Homarus gammarus nudivirus) (YP_010087670.1)	
CmNV_30	38,180	38,934	+	251	hypothetical protein KM727_gp31 (Homarus gammarus nudivirus) (YP_010087671.1)	
CmNV_31	39,020	39,714	+	231	hypothetical protein KM727_gp32 (Homarus gammarus nudivirus) (YP_010087672.1)	
CmNV_32	39,705	40,444	+	246	PmV-like protein (Homarus gammarus nudivirus) (YP_010087673.1)	
CmNV_33	40,531	41,633	+	367	p-loop NTPase (Homarus gammarus nudivirus) (YP_010087674.1)	
CmNV_34	41,687	42,612	+	308	hypothetical protein KM727_gp35 (Homarus gammarus nudivirus) (YP_010087675.1)	
CmNV_35	42,619	44,375	+	585	hypothetical protein KM727_gp36 (Homarus gammarus nudivirus) (YP_010087676.1)	
CmNV_36	44,330	44,643	-	104	hypothetical protein KM727_gp37 (Homarus gammarus nudivirus) (YP_010087677.1)	
CmNV_37	44,618	44,826	+	69	hypothetical protein PmNV_051 (Penaeus monodon nudivirus) (YP_009051889.1)	Ac92-like protein
CmNV_38	44,847	45,364	+	172	hypothetical protein KM727_gp39 (Homarus gammarus nudivirus) (YP_010087679.1)	LEF-5
CmNV_39	46,080	46,729	+	216	hypothetical protein KM727_gp41 (Homarus gammarus nudivirus) (YP_010087681.1)	
CmNV_40	46,737	47,650	+	304	integrase (Homarus gammarus nudivirus) (YP_010087682.1)	
CmNV_41	47,682	48,532	+	283	VLF-1 (Homarus gammarus nudivirus) (YP_010087683.1)	
CmNV_42	48,543	48,910	-	122		
CmNV_43	48,980	50,133	-	384	hypothetical protein KM727_gp94 (Homarus gammarus nudivirus) (YP_010087734.1)	
CmNV_44	50,276	51,675	-	466		
CmNV_45	52,559	52,812	-	84		
CmNV_46	52,816	54,416	-	533	LEF-9 (Homarus gammarus nudivirus) (YP_010087686.1)	
CmNV_47	54,427	55,265	+	279	38K protein (Homarus gammarus nudivirus) (YP_010087687.1)	
CmNV_48	55,271	55,533	-	87	hypothetical protein KM727_gp48 (Homarus gammarus nudivirus) (YP_010087688.1)	
CmNV_49	55,538	56,244	+	235	hypothetical protein KM727_gp49 (Homarus gammarus nudivirus) (YP_010087689.1)	
CmNV_50	56,229	56,626	+	132	hypothetical protein KM727_gp50 (Homarus gammarus nudivirus) (YP_010087690.1)	GbNV_gp51-like
CmNV_51	56,625	56,974	-	116	hypothetical protein PmNV_063 (Penaeus monodon nudivirus) (YP_009051901.1)	
CmNV_52	57,089	57,429	-	113		
CmNV_53	57,473	58,803	-	443	p-loop NTPase (Homarus gammarus nudivirus) (YP_010087693.1)	TK1
CmNV_54	58,872	60,178	+	435	hypothetical protein KM727_gp54 (Homarus gammarus nudivirus) (YP_010087694.1)	
CmNV_55	60,317	60,600	+	94		
CmNV_56	61,344	63,214	+	623	ODV-E66 (Penaeus monodon nudivirus) (YP_009051874.1)	
CmNV_57	63,746	64,575	-	276		IAP
CmNV_58	65,340	65,920	-	193	hypothetical protein PmNV_067 (Penaeus monodon nudivirus) (YP_009051905.1)	
CmNV_59	66,051	66,622	-	190	hypothetical protein KM727_gp58 (Homarus gammarus nudivirus) (YP_010087698.1)	
CmNV_60	66,657	67,597	-	313	hypothetical protein KM727_gp59 (Homarus gammarus nudivirus) (YP_010087699.1)	
CmNV_61	67,580	68,187	-	202	hypothetical protein KM727_gp60 (Homarus gammarus nudivirus) (YP_010087700.1)	
CmNV_62	68,259	69,631	-	457	hypothetical protein KM727_gp61 (Homarus gammarus nudivirus) (YP_010087701.1)	
CmNV_63	69,724	71,798	-	691	P74 (Homarus gammarus nudivirus) (YP_010087703.1)	PIF-0
CmNV_64	71,846	72,693	+	282		
CmNV_65	72,750	73,375	+	208	hypothetical protein KM727_gp65 (Homarus gammarus nudivirus) (YP_010087705.1)	
CmNV_66	73,336	75,113	+	592	helicase 2 (Homarus gammarus nudivirus) (YP_010087706.1)	
CmNV_67	75,187	76,055	+	289	hypothetical protein KM727_gp67 (Homarus gammarus nudivirus) (YP_010087707.1)	
CmNV_68	76,061	76,707	-	215	hypothetical protein KM727_gp68 (Homarus gammarus nudivirus) (YP_010087708.1)	
CmNV_69	76,697	78,657	+	653	helicase 2 (Homarus gammarus nudivirus) (YP_010087709.1)	
CmNV_70	78,639	84,124	+	1828	hypothetical protein KM727_gp70 (Homarus gammarus nudivirus) (YP_010087710.1)	
CmNV_71	84,170	85,047	+	292	hypothetical protein KM727_gp72 (Homarus gammarus nudivirus) (YP_010087712.1)	
CmNV_72	85,039	85,598	+	186	hypothetical protein KM727_gp73 (Homarus gammarus nudivirus) (YP_010087713.1)	
CmNV_73	85,616	87,168	-	517	hypothetical protein KM727_gp74 (Homarus gammarus nudivirus) (YP_010087714.1)	
CmNV_74	87,163	87,521	-	119	hypothetical protein KM727_gp75 (Homarus gammarus nudivirus) (YP_010087715.1)	
CmNV_75	87,520	88,037	+	172	Ac81-like protein (Homarus gammarus nudivirus) (YP_010087716.1)	
CmNV_76	88,025	89,913	+	629	hypothetical protein KM727_gp77 (Homarus gammarus nudivirus) (YP_010087717.1)	
CmNV_77	89,921	90,351	+	143	Ac68-like protein (Homarus gammarus nudivirus) (YP_010087718.1)	PIF-6
CmNV_78	90,389	91,647	-	419	hypothetical protein KM727_gp79 (Homarus gammarus nudivirus) (YP_010087719.1)	
CmNV_79	91,740	92,467	+	242	VLF-1 (Homarus gammarus nudivirus) (YP_010087720.1)	
CmNV_80	92,474	93,867	-	464	LEF-4 (Homarus gammarus nudivirus) (YP_010087721.1)	
CmNV_81	93,888	94,300	-	137	hypothetical protein KM727_gp82 (Homarus gammarus nudivirus) (YP_010087722.1)	
CmNV_82	94,298	95,016	-	239	PIF-3 (Homarus gammarus nudivirus) (YP_010087723.1)	
CmNV_83	95,017	98,831	-	1271	helicase (Homarus gammarus nudivirus) (YP_010087724.1)	
CmNV_84	98,830	99,518	+	229	ODV-E28 (Penaeus monodon nudivirus) (YP_009051934.1)	PIF-4
CmNV_85	99,512	100,257	-	248	hypothetical protein KM727_gp86 (Homarus gammarus nudivirus) (YP_010087726.1)	
CmNV_86	100,256	101,100	+	281	esterase (Homarus gammarus nudivirus) (YP_010087727.1)	GbNV_gp19-like
CmNV_87	101,113	102,344	-	410	hypothetical protein KM727_gp88 (Homarus gammarus nudivirus) (YP_010087728.1)	GbNV_gp67-like
CmNV_88	102,446	102,744	-	99	11K virion structural protein (Homarus gammarus nudivirus) (YP_010087729.1)	
CmNV_89	102,869	103,191	-	107	hypothetical protein KM727_gp90 (Homarus gammarus nudivirus) (YP_010087730.1)	
CmNV_90	103,184	103,956	+	257	hypothetical protein KM727_gp91 (Homarus gammarus nudivirus) (YP_010087731.1)	
CmNV_91	104,012	105,081	+	356	death-associated inhibitor of apoptosis 1 (Homarus gammarus nudivirus) (YP_010087732.1)	IAP
CmNV_92	105,794	106,290	-	165		
CmNV_93	106,479	106,957	-	159		
CmNV_94	107,140	108,752	-	537	polyhedrin, partial (Penaeus monodon nucleopolyhedrovirus) (AET06106.1)	
CmNV_95	109,297	110,303	-	335		
CmNV_96	111,058	111,419	+	120		
CmNV_97	112,295	113,013	-	239	hypothetical protein KM727_gp96 (Homarus gammarus nudivirus) (YP_010087736.1)	
CmNV_98	113,157	113,803	+	215	hypothetical protein KM727_gp97 (Homarus gammarus nudivirus) (YP_010087737.1)	
CmNV_99	45,460	45,651	+	64		p6.9

**Table 3:** List of identified core genes across 15 nudiviruses. Numbers represent open reading frame (ORF) numbers for each of the nudiviruses. Genes (proteins) that were not reported in the proteomes on NCBI, but that were subsequently found in the current or previous studies are indicated and referred to in the footnote.

Gene	Description	CcNV	CmNV	DhNV	DiNV	ENV	GbNV	HgNV	HzNV-1	HzNV-2	KNV	MNV	OrNV	PmNV	TNV	ToNV
11K-like	Occlusion body component	101	88	101	17	37	95	89	124	25	44	40	41	100	32	28
38K	Nucleocapsid protein	61	47	60	46	14	1	47	10	108	20	14	87	59	59	63
ac81	Nucleocapsid envelopment	83	75	81	62	4	14	76	33	96	7	4	4	86	4	123
dnapol	DNA polymerase	1	1	1	65	1	12	1	131	18	10	1	1	5	1	12
fen-1	FEN-1/FLAP endonuclease	12	13	12	100	56	65	13	68	70	68	62	16	20	17	1
GbNV_gp19-like	Unknown	98	86	99	22	32	19	87	30	99	39	35	47	98	38	27
GbNV_gp51-like	Unknown	64	50	63	34	22	51	50	^a^	79	30	26	61	62	48	19
GbNV_gp67-like	Unknown	99	87	100	102	54	67	88	122	27	66	60	18	99	15	6
helicase	DNA helicase	93	83	95	12	42	88	84	104	38	49	45	34	94	26	118, 55
helicase-2	DNA helicase	77, 89	66, 69	87, 90	90	65	46	66, 69	60	76	78	71	108	76, 79	74	105
integrase	DNA processing	56	40	54	39	18	57	42	144	8	25	21	75	55	54	43
lef-4	RNA polymerase subunit	90	80	92	18	36	96	81	98	43	43	39	42	91	33	25
lef-5	Transcription initiation factor	53	38	51	25	^c^	85	39	101	40	^c^	^c^	52	52	40	50, 66
lef-8	RNA polymerase subunit	15	16	15	32	24	49	16	90	51	33	29	64	23	46	88
lef-9	RNA polymerase subunit	60	46	56	57	9	24	46	75 ^a,^*	63 ^a,^*	4	8	96	58	65	131
p33_ac92	Sulfhydryl oxidase	4	4	3	87	67	7	4	13	104	82	74	113	8	75	99
p47	RNA polymerase subunit	8	8	7	104	53	69	8	75 ^a,^*	63 ^a,^*	64	58	20	14	13	115
p6.9	Nucleocapsid packaging/assembly	106 ^b^	99 ^b^	-	1	51	72	40	142 ^a^	^a^	61	56	22	^a^	11	51
pif-0/p74	Per os infectivity factor	74	63	71	74	80	45	63	11	106	95	88	126	72	88	45
pif-1	Per os infectivity factor	35	27	39	35	21	52	28	55	82	29	25	60	39	49	69
pif-2	Per os infectivity factor	9	9	8	101	55	66	9	123	26	67	61	17	15	16	7
pif-3	Per os infectivity factor	92	82	94	91	64	3	83	88	53	77	70	107	93	73	13
pif-4	Per os infectivity factor	94	84	96	11	43	87	85	103	39	51	46	33	96	25	119
pif-5/odv-e56	Per os infectivity factor	6	6	5	6, 85	69	5	6	76	62	56, 84	51, 76	115	10	77	102, 74, 96
pif-6	Per os infectivity factor	85	77	79	36	20	55	78	74	64	28	24	72	88	51	56
vlf-1	Very late gene expression factor	59	41	55	9	45	80	43	121	28	53	48	30	56	24	65
vp39	Major capsid protein	14	15	14	99	57	64	15	89	52	69	63	15	22	18	87
vp91/p95	Nucleocapsid protein	5	5	4	92	63	2	5	46	89	76	69	106	9	72	16, 83

^a^ Bezier et al., 2015, ^b^ manual blast search, ^c^ vgas gene prediction, * p47 and lef-9 genes are fused (Bezier et al., 2015; Holt et al., 2019).

## Data Availability

Original slides used for this paper are stored together with biological material embedded in wax and epoxy resin in the Cefas Weymouth Lab. The type material is stored as RA16005, sample 44 (*Crangon crangon*) and RA14060, sample 161 (*Carcinus maenas*). The CcNV and CmNV genome sequences have been deposited to GenBank under Accession Numbers MZ311577 and MZ311578.

## Supplementary Materials

The following are available online at https://www.mdpi.com/article/10.3390/v13091694/s1, Table S1. Full annotation table for CcNV, detailing the position of predicted gene sequences and their annotations based on blastp similarity searches (best hit; *p* < 0.001; NCBI nr protein database) and InterProScan results. Table S2. Full annotation table for CmNV, detailing the position of predicted gene sequences and their annotations based on blastp similarity searches (best hit; *p* < 0.001; NCBI nr protein database) and InterProScan results. Table S3. Tandem repeats within the CcNV genome. Table S4. Tandem repeats within the CmNV genome. Table S5. Orthogroups identified by comparing the proteomes of 15 nudiviruses and AcMNPV (NC_001623.1) as outgroup. Analysis was conducted using OrthoFinder v2.3.11 and the following parameters: -A muscle -M msa -T raxml. Gene description annotations for each virus were obtained from corresponding NCBI records (see main text for Accession Numbers).
